# International Medical Annual and Practitioner’s Index

**Published:** 1896-04

**Authors:** 


THE INTERNATIONAL MEDICAL ANNUAL AND PRACTITIONER'S INDEX. A work of reference for medical practitioners. 1896. Fourteenth year. New York: E. B. Treat, 5 Cooper Union. Chicago, 199 Clark Street. Price, $2.75.

The publishers of this work have sought to make the present work quite equal to any of its predecessors, if it does not surpass them.

Looking over the book we meet with a most valuable discussion upon

Chloroform and its dangers, upon the best method of resuscitating those who have been put in jeopardy by its use.

In the article upon antiseptics, we observe a strong recommendation of the value of Acetanilide as a substitute for Iodoform.

In skin diseases the treatment with electricity in currents of great frequency and high tension is recommended and permanent cures reported.

Quite a large number of the newer drugs introduced into medicine from time to time are mentioned with new points in their action and their management reported.

Under Mercury attention is called to the value of blue-pill in heart disease. A case is reported of a man with valvular disease of the heart who was kept alive for ten years, by the continual taking of blue-pills. It is estimated that the patient took twenty thousand grains of Blue Mass in that time, and yet he was never salivated or purged.

Under Potassium Permanganate, a case is reported of poisoning with an over-dose of Morphia, which was successfully antidoted by the Permanganate.

Rhus-toxicodendron is mentioned and its value in treating various morbid conditions "from noctural incontinence of urine to pemphigus," is asserted.

Under "Thyroid Extract" a case of acute glycosuria is reported from taking tablets of this preparation daily to cure psoriasis.

On page 72 may be found an excellent article on the "Diagnosis of Toothache and Neuralgia of Dental Origin, by Henry Sewill."

There is a timely article upon the "remedial value of cycling."

There is also a valuable article upon "The Sensory Distribution of Nerve Roots," illustrated by colored plates.

Angio-neurosis, illustrated by photographs, life assurance, abdominal surgery, mammary cancer, illustrated with colored drawings; Antitoxin treatment of diphtheria, diseases of the ear—an elaborate article with colored plates—diseases of the eye, obstinate hiccough, diseases of the larynx, orthopædics—with many photographic illustrations—prostatic diseases, tuberculosis and typhoid fever, are among the articles noticed by the writer as being of particular interest because of the very latest developments in their pathology and treatment. The Rœntgen Rays are spoken of, and considerable cold water thrown upon those whose optimism has led them to some extravagant expectations of what may be accomplished by these extraordinary phenomena.

The foregoing account of the contents of this book will enable the reader to appreciate the very wide horizon upon the latest medical discoveries is afforded him.

